# Studies of the Immunomodulatory Activity of Polysaccharides from the Stem of *Cynomorium songaricum* Based on Intestinal Microbial Analysis

**DOI:** 10.3390/molecules29010143

**Published:** 2023-12-26

**Authors:** Tong Lv, Jiarong Chen, Zhongmei He, Weijia Chen, Ying Zong, Rui Du

**Affiliations:** 1 College of Traditional Chinese Medicine, Jilin Agricultural University, Changchun 130118, China; 18804379689@163.com (T.L.); chenjiarong0121@163.com (J.C.); heather78@126.com (Z.H.); chenweijia_jlau@163.com (W.C.); 2 Jilin Provincial Engineering Research Center for Efficient Breeding and Product Development of Sika Deer, Changchun 130118, China

**Keywords:** *Cynomorium songaricum* stem, polysaccharide, isolation and purification, immunological effects, intestinal flora

## Abstract

Polysaccharides are the main effective components of *Cynomorium songaricum*’s stem that perform biological activities and have positive impacts on immune enhancement. In this study, the polysaccharide CSP-III of *Cynomorium songaricum*’s stem was isolated using a DEAE-52 cellulose column through Sephadex G-100 gel column chromatography. Upon analysis, the monosaccharide composition of CSP-III included Mannose (Man), Glucuronic acid (GlcA), Galacturonic acid (GalA), Rhamnose (Rha), Glucose (Glc), Galactose (Gal), and Arabinose (Ara), at a molar ratio of 0.01:0.11:0.03:0.57:0.02:0.32:1. The molecular weight of CSP-III was 4018234 Da. Meanwhile, the capacity of CSP-III, at various concentrations, to stimulate the proliferation of mouse spleen lymphocytes in vitro was compared, and the influence of CSP-III on cell proliferation was examined using RAW264.7 mouse mononuclear macrophages as a model. The influence of CSP-III on the expression of important phosphorylating proteins in the MAPK signaling pathway was initially analyzed by Western blotting. In RAW264.7 cells, CSP-III promoted the phosphorylation of JNK proteins, which thus activated the MAPK signaling cascade and exerted immunomodulatory effects. Moreover, according to in vivo studies using cyclophosphamide (CTX)-induced immunosuppression mouse models, CSP-III improved the CTX-induced histopathological damage, promoted T and B lymphocyte proliferation, upregulated CD4+ and CD8+ T-lymphocyte counts in the spleen, increased the serum levels of IgG and IgM, and activated three essential proteins of the MAPK signaling pathway. As revealed by analysis of intestinal flora, CSP-III improved the immune function by maintaining the homeostasis of the bacterial flora by boosting the relative abundances of some beneficial bacterial groups, such as Bacteroidetes, Desmodium, and Actinomyces, and reducing the relative abundance of Aspergillus phylum. Through in vitro and in vivo experiments, our present study demonstrates that polysaccharides from the stem of *Cynomorium songaricum* possess strong immunoregulatory effects. Findings in this work provide theoretical support for the potential application of *Cynomorium songaricum* in the field of health food.

## 1. Introduction

The dry, fleshy stem of *Cynomorium* *conarium*, also called “Suoyang” in China, has been widely used as a traditional Chinese medicine to treat a variety of diseases including premature ejaculation, impotence, low libido, nocturnal colic, and gastric ulcers [1]. The plant *Cynomorium songaricum* is a perennial fleshy parasitic herb, mostly found in desert areas, and its fleshy stems are of medicinal and edible value. Typically, Inner Mongolia is the main provider of wild resources of *Cynomorium songaricum*, and generates approximately 70 percent of the country’s total output. The artificial planting and traditional technology of *Cynomorium songaricum*, which has been developed into the primary planting bases in Su’nan of Gansu and Ayuqi of Inner Mongolia, has been extensively studied in China since 2004 [2,3]. Aside from China, *Cynomorium songaricum* can be found in Central Asia, Iran, and Mongolia [4]. Islamic people use *Cynomorium songaricum* to treat hemorrhoids and nasal and uterine hemorrhages, while in traditional medicine from North Africa it is believed to be an aphrodisiac, spermatogenic, tonic, and astringent. Medieval Arabs thought that *Cynomorium songaricum* can treat blood disorders, digestive disorders, and reproductive problems. In accordance with recent scientific reports, *Cynomorium songaricum* contains a variety of biologically active compounds that can improve the sexual function, immunity, and hypoxia resistance of the body, and produce other pharmacological effects like reducing stress and fatigue.

Numerous herbal remedies consist of polysaccharides, a type of complex carbohydrates that are connected by glycosidic linkages and have an array of biological functions [5,6]. According to current pharmacological studies, polysaccharides exhibit a range of pharmacological effects, including antitumor, antioxidant, anticoagulant, antiviral, hypoglycemic, hypolipidemic, and immunomodulatory effects, all of which are crucial for the growth and development of living organisms [7]. Moreover, polysaccharides have steadily developed into a hub for medication development and application research [8]. Notably, polysaccharides are the primary active ingredients of *Cynomorium songaricum* and probable active sites of significant research significance.

The intestine is a crucial organ for digestion and absorption, which plays a role in the body’s circulatory metabolism. Moreover, it also acts as a natural barrier for defense against the invasion of harmful pathogenic bacteria, viruses, and other microorganisms [9,10,11]. Intestinal flora is an essential link in the interaction between polysaccharides and the human body. Apart from contributing to energy metabolism and the immune response of the body, polysaccharides can be converted by the intestinal flora into short-chain fatty acids and carbon monoxide while still having an effect on the diversity, species, and ratio of the host’s intestinal flora [12,13,14,15,16]. The correct growth and maturity of the immune system and mucosal immune system depend on the changes in intestinal flora.

Macrophages and lymphocytes are frequently selected as the best cellular models for analyzing immunity to bioactive components, since studies have demonstrated that plant polysaccharides are strongly related to immunomodulatory effects. It has been demonstrated that *Cynomorium songaricum*’s crude polysaccharides promote B lymphocyte proliferation [17]. Moreover, as revealed by a study of the in vitro immunoreactivity of two newly purified fractions of *Cynomorium songaricum* polysaccharides, both fractions have an inhibitory effect on the level of cytokine IL-2 [18]. Furthermore, immunosuppressive mouse model analysis results suggest that *Cynomorium songaricum* polysaccharides may improve the immune function in the body by promoting macrophage proliferation, which is achieved via its advantageous immunomodulatory effect on macrophages RAW264.7 within a specific concentration range. However, the immunoregulatory effects of *Cynomorium songaricum* polysaccharides should be further explored.

In this study, homogeneous polysaccharides were produced by extracting and purifying *Cynomorium songaricum* stem polysaccharides (CSPs). Meanwhile, in vivo and in vitro experiments were performed using RAW264.7 cells and mice treated with the immunosuppressive drug cyclophosphamide as models, respectively. Thereafter, the experimental results were combined with intestinal flora examination to investigate the immune modulating mechanism of CSPs.

## 2. Results

### 2.1. Isolation and Purification of Polysaccharides

**Figure 1:**
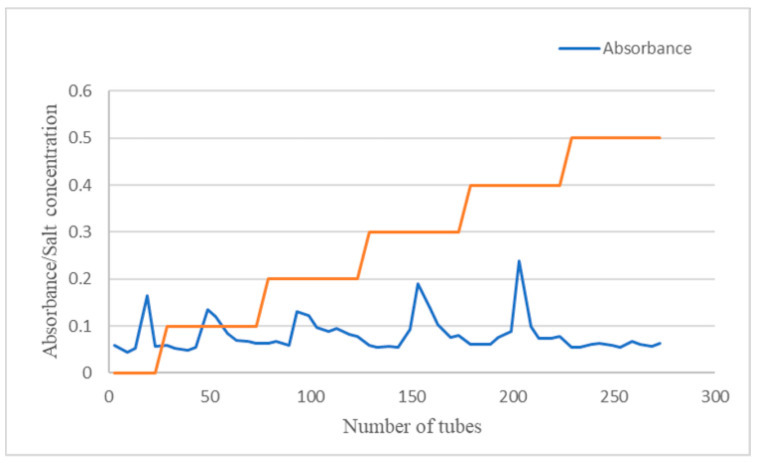
Separation of CSP via the DEAE-52 fiber column method.

**Figure 2:**
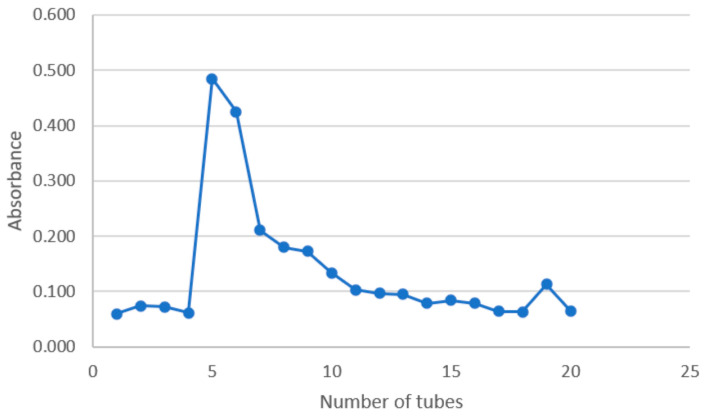
Separation of CSP via the Sephadex G-100 gel method.

Through anion-exchange chromatography and gel exclusion chromatography using DEAE cellulose and Sephadex-G100 columns, respectively, the CSP-I separated from *Cynomorium songaricum*’s stem was graded. After the elimination of proteins and ionic compounds with a DEAE cellulose column, the recovered polysaccharides were purified by deionized water elution using Sephadex-G100. Moreover, five elution sites, CSP-II-1, CSP-II-2, CSP-II-3, CSP-II-4, and CSP-II-5, were produced via gradient elution with water and NaCl (Table 1, Figure 1). However, the polysaccharide contents of CSP-II-1, CSP-II-2, CSP-II-4, and CSP-II-5 were relatively low in this experiment; only the polysaccharide CSP-II-3 was further purified by Sephadex G-100 gel chromatography (Figure 2), and the purified polysaccharide CSP-II-3 was named CSP-II, with a yield of 7% (dry weight of CSP-II/dry weight of CSP-I). Thereafter, CSP-II was purified by Sephadex G-100 gel column chromatography to give an elution site named CSP-III, with a yield of 44.74% (dry weight of CSP-III/dry weight of CSP-II).

### 2.2. Monosaccharide Composition

**Figure 3:**
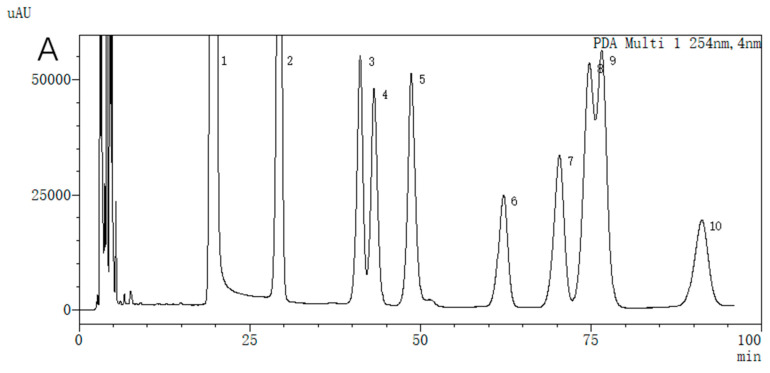

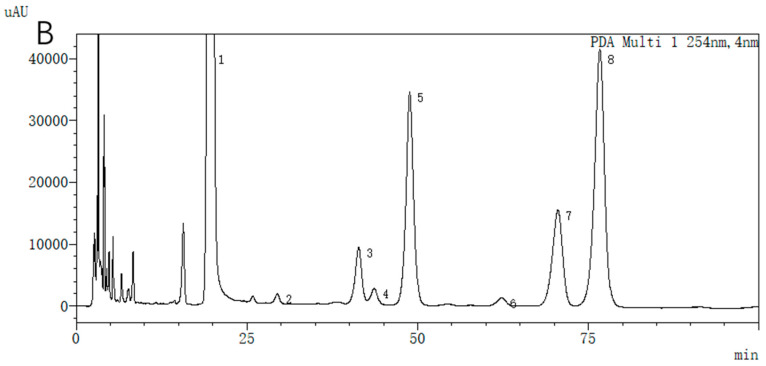
HPLC chromatogram showing the monosaccharide composition ((**A**) Mixed standards; (**B**) CSP-III). 1—PMP, 2—Man, 3—GlcA, 4—GalA, 5—Rha, 6—Glc, 7—Gal, 8—Xyl, 9—Ara, 10—Fuc.

The polysaccharide CSP-III displayed seven peaks on an HPLC graph, as illustrated in Figure 3. The constituent sugars included Man, GlcA, GalA, Rha, Glc, Gal, and Ara at a molar ratio of 0.01: 0.11: 0.03: 0.57: 0.02: 0.32:1 determined by the external standard method.

### 2.3. Molecular Weight Determination Results

**Figure 4:**
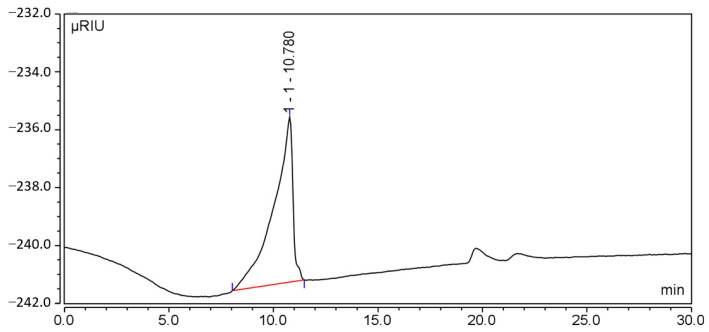
Determination results of CSP-III. Red line: baseline, used to calculate molecular weight.

High-performance gel molecular-exclusion chromatography was conducted to measure the molecular weight. Figure 4 displays the resulting chromatogram. The molecular weight and PDI of the total peak were determined to be 4,018,234 Da and 10.62, respectively.

### 2.4. In Vitro Experiments

#### 2.4.1. Spleen Lymphocyte Proliferation Assay Results

**Figure 5:**
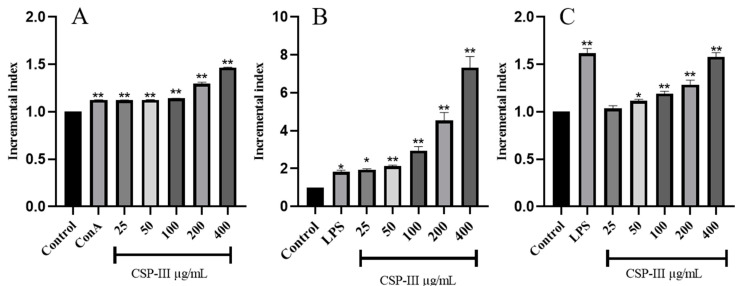
Effect of CSP-III on the proliferation of T (**A**), B lymphocytes (**B**), and macrophages (**C**). Compared with the blank group, * *p* < 0.05, ** *p* < 0.01.

The proliferation of mouse T and B lymphocytes was considerably boosted by CSP-III in the concentration range of 25–400 μg/mL, with ConA and LPS being the positive controls, respectively, as demonstrated in Figure 5A,B. The proliferation index rose in a dose-dependent manner as the drug concentration increased (*p* < 0.05, *p* < 0.01). Such results suggested that the proliferation of mouse T and B lymphocytes might be aided by polysaccharide CSP-III, and the growth trend was more pronounced.

#### 2.4.2. RAW264.7 Macrophage Proliferation Assay Results

As demonstrated in Figure 5C, there was a substantial difference in the proliferation index between LPS group and blank group, demonstrating that LPS stimulated macrophage proliferation. To be specific, the proliferation index increased with the increasing concentration of CSP-III to 25, 50, 100, 200, and 400 μg/mL. At 400 μg/mL, the proliferation index approached that of the LPS group, suggesting that the polysaccharide CSP-III extremely significantly (*p* < 0.01, *p* < 0.05) promoted the proliferation of RAW264.7 macrophages.

#### 2.4.3. Impact of CSP-III on the Protein Expression of the MAPK Signaling Pathway

**Figure 6:**
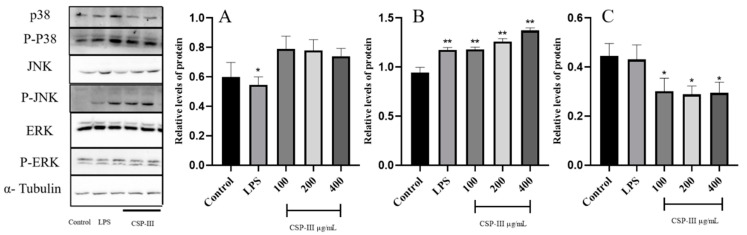
Effect of CSP-III on the protein expression of the MAPK signaling pathway ((**A**) p-P38; (**B**) p-JNK; (**C**) p-ERK). Compared with the blank group, * *p* < 0.05, ** *p* < 0.01.

Three proteins were detected in the MAPK pathway, as shown in Figure 6. It was discovered that CSP-III promoted the phosphorylation of JNK proteins and activated the JNK signaling pathway. The protein expression was gradually upregulated as CSP-III concentration increased within the concentration range (*p* < 0.05 or *p* < 0.01).

### 2.5. In Vivo Experiments

#### 2.5.1. Spleen Lymphocyte Proliferation Assay Results

**Figure 7:**
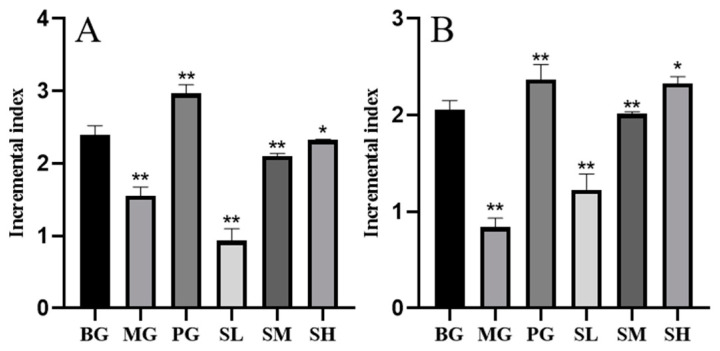
Effect of polysaccharide CSP-III on mouse spleen lymphocytes ((**A**) T lymphocytes; (**B**) B lymphocytes). Compared with the blank group, * *p* < 0.05, ** *p* < 0.01.

According to Figure 7, mice in the MG group exhibited considerably lower T and B lymphoid value-added indices than those in the BG group, while mice in the PG group had significantly higher lymphocyte value-added indices (*p* < 0.01). Additionally, all the other dosing groups had increased proliferation indices of T and B lymphocytes, with the exception of the low-dose group. This indicated that the polysaccharide CSP-III had a pro-proliferative effect on T and B lymphocytes in immunosuppressive mice.

#### 2.5.2. T-Lymphocyte Subsets Detected by Flow Cytometry

**Figure 8:**
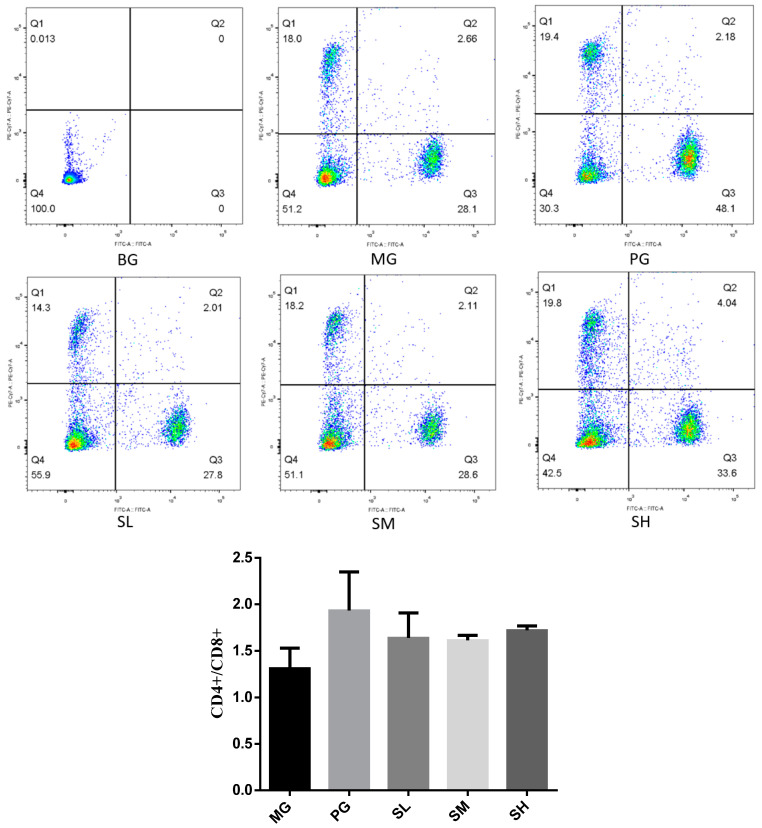
T-lymphocyte subsets detected via flow cytometry.

According to Figure 8, the CD4+/CD8+ ratio of the MG group was considerably lower than that of the BG group, even though that of the positive group was greater than that of the normal group. Moreover, the modulating effect of polysaccharides was more pronounced in the high-dose group, meanwhile, immunosuppressive mice treated with high-dose CSP-III showed an elevated CD4+/CD8+ ratio, which was significantly different from that of model group. This suggested that the CTX-induced decrease in CD4+ T lymphocytes was reversed by the action of CSP-III.

#### 2.5.3. Impact of CSP-III on the IgG and IgM Serum Levels in Mice

**Figure 9:**
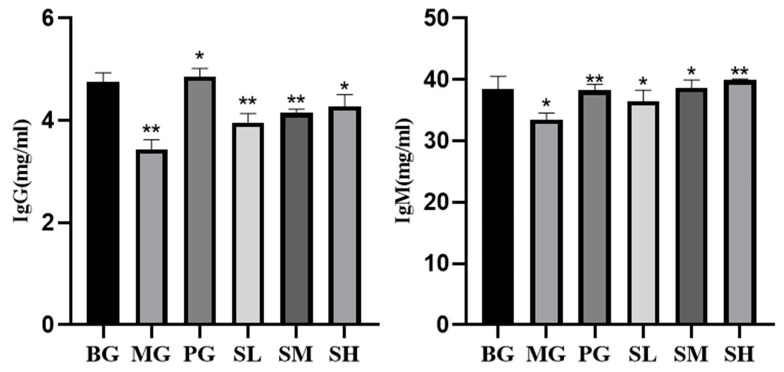
Effect of polysaccharide CSP-III on IgG and IgM serum levels in mice. Compared with the blank group, * *p* < 0.05, ** *p* < 0.01.

IgG and IgM contents in the MG group were considerably lower than those in the BG group, as demonstrated in Figure 9 (*p* < 0.01). Additionally, the serum levels of IgG and IgM in the mice of all dosing groups were higher than those in the MG group, and those in the high-dose group were significantly higher and more similar to those in the PG group. This revealed that polysaccharide CSP-III improved immunity by raising the serum levels of IgG and IgM in mice.

#### 2.5.4. Effect of CSP III on the Morphological Structure of Mouse Mesenteric Lymph Nodes and Spleen

**Figure 10:**
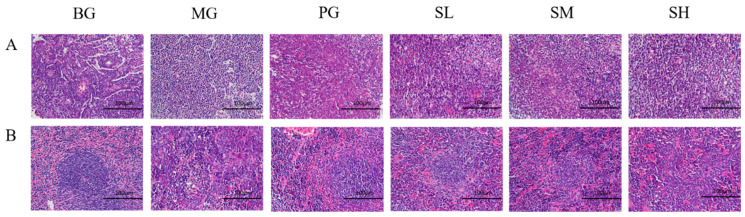
HE staining results ((**A**) mesenteric lymph nodes; (**B**) spleen).

As demonstrated in Figure 10A, in comparison with the BG group, the lymph nodes in the MG group generally exhibited atrophy, the distribution of lymphoid tissues and lymphoid nodules in the lymph nodes was significantly reduced, and no germinal centers were observed in several lymphoid nodules. These findings implied that the maturation of lymphocytes in lymph nodes and their capacity to elicit an immune response were both significantly reduced. In contrast, the polysaccharide administration group was able to reverse the effects of CTX by increasing the distribution of lymphoid tissues and the number of lymphoid nodes in lymph nodes. Similar to the polysaccharide SM group, the PG group experienced an increase in lymphoid nodes in lymph nodes.

Figure 10B illustrates the distribution of both red and white medulla in the spleens of the BG group. As observed, there were periarterial lymphatic sheaths and lymphoid nodules in the white medulla, whereas there were birth centers and stellate phenomena in the lymphoid nodules. Further, there was poor demarcation between the red and white medullas in the spleens of the MG group, and the amount of white medulla components was significantly reduced. The white medulla also included few lymphoid nodules and birth centers. In comparison, the red medulla included a large number of cellular components, which frequently displayed the red medullary stagnation phenomenon. The administration of polysaccharides had a more noticeable improvement effect on the spleens. To be specific, polysaccharides increased the distribution of white medulla, the distribution of periarterial lymphatic sheaths, the distribution of lymphoid nodules, and the overall immune function of the mouse spleens. The improvement effect of the polysaccharide group was slightly stronger than that of the PG group.

#### 2.5.5. Effect of CSP III on the Expression of MAPK Signaling Pathway-Related Proteins in the Spleen of Mice

**Figure 11:**
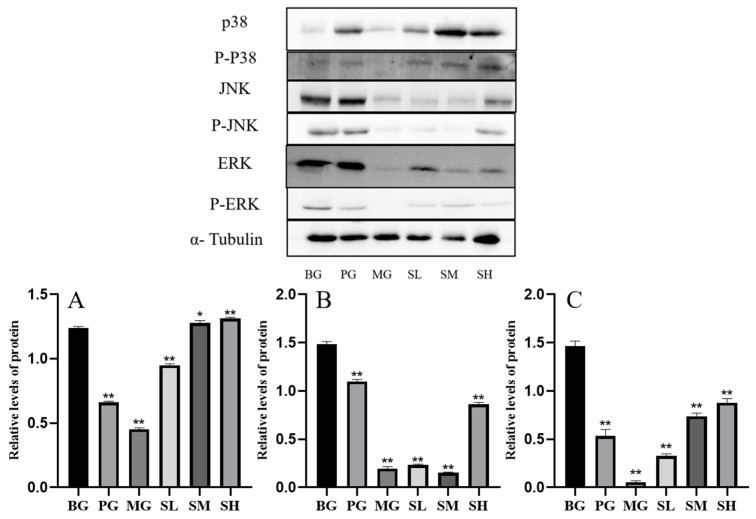
Expression of MAPK signaling pathway-related proteins ((**A**) p-P38; (**B**) p-JNK; (**C**) p-ERK). Compared with the blank group, * *p* < 0.05, ** *p* < 0.01.

Figure 11 shows the detection results of three proteins in the MAPK pathway. As observed, the p38 signaling pathway was significantly activated after polysaccharide treatment, and the protein expression tended to rise in a dose-dependent manner. What is more, the ERK and JNK signaling pathways were also significantly activated by polysaccharide treatment, and the protein expression increased in a dose-dependent manner.

#### 2.5.6. Analysis of Intestinal Flora Data in Mice

**Figure 12:**
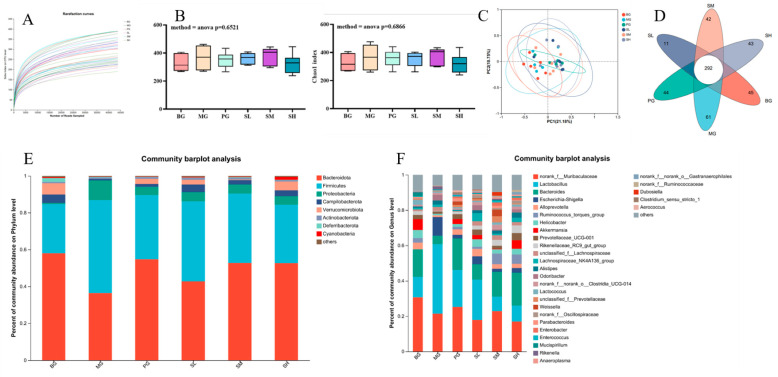
Analysis chart of intestinal flora ((**A**) sparse curve; (**B**) Alpha diversity index; (**C**) distance matrix and PCoA analysis; (**D**) Wayne chart; (**E**) phyla-level flora abundance; (**F**) genus-level flora abundance).

As shown in Figure 12A, the dilution curve responded to the depth of sequencing that captured rare lineage types and the majority of the diversity. It was observed that, the dilution curve gradually flattened out, indicating that the majority of the microbial diversity was obtained and that a sufficient quantity of sequencing data were available. Alpha diversity counts the number of taxa in each sample and determines if they are spread evenly. Figure 12B illustrates how the taxonomic diversity of the high-dose group came close to matching that of the control group following the intervention.

Diversity compares the make-up of microbial communities and assesses the variations among microbial communities. In this study, the species composition of the groups administered at each dose, the positive group and the model group were either inclusive or non-intersecting, indicating an increase in species diversity among various habitats after drug administration. As observed from Figure 12C, the species compositions of the blank group and model group were inclusive; in other words, the model group showed a decrease in species diversity compared with normal group.

Venn diagrams were plotted to identify the traits and shared taxa of various treatment groups. According to Figure 12D, there were altogether 292 OTUs in the intestinal flora of all the six groups, including 45 OTUs in the BG group, 61 OTUs in the MG group, 11 OTUs in the SL group, 42 OTUs in the SM group, 43 OTUs in the SH group, and 44 OTUs in the PG group. There were more OTUs in the model group than in the blank group, which might be because the intestinal flora of model mice was dysbiotic, thus causing an increase in several microbial species. Clearly, after drug administration, the intestinal flora of the positive drug group and the medium- and high-dose treatment groups was closer to that of control group.

Bacteroidetes, Firmicutes, Proteobacteria, Campliobacterota, Verrucomicrobia, Actinobacteria, Deferribacteres, Cyanobacteria, and other phyla are presented at the phylum level in Figure 12E, with anamorphs and thick-walled phyla composition having the highest proportion. As observed, drug administration reversed the changes in CTX-induced abundances of bacterial groups and returned them to normal levels. Compared with the BG group, the relative abundance of Ascomycetes in the MG group was higher and those of thick-walled Bacteria and Anabacteria were significantly lower.

A total of 30 colonies were identified at the genus level, as shown in Figure 12F. The genera Muribaculaceae, Lactobacillus, Bacteroides (Bacteroides), Escherichia-Shigella (Escherichia-Shigella), and alloprevotella were among the principal components. When comparing the MG group with the BG group, the relative abundances of genera Shigella and Lactobacillus increased, while those of genera Muribaculaceae and Bacteroides declined. After drug administration, the relative abundances of Mycobacterium and Muribaculaceae species in each dosage group reverted to normal levels, with the medium- and high-dose groups showing more noticeable effects.

## 3. Discussion

The monosaccharide composition is the most fundamental and core research object in plant polysaccharide research. It is closely related to the physicochemical properties, structural characteristics, and structure–activity relationship of polysaccharides [19]. In this study, the dry and fleshy stems of *Cynomorium songaricum* were used to isolate and purify CSP-III, whose molecular weight was determined to be 4,018,234 Da and the monosaccharide composition included Man, GlcA, GalA, Rha, Glc, Gal, and Ara at a molar ratio of 0.01:0.11:0.03:0.57:0.02:0.32:1. The molecular weight of polysaccharides has a significant impact on their biological activity and is one of the key indicators for evaluating the quality of polysaccharides [20]. Researchers have reported that the molecular weight of polysaccharides obtained from different sources of *Cynomorium songaricum* or from the same source under different extraction conditions and purification processes varies. Wang Junlong et al. [21] separated a water-soluble *Cynomorium songaricum* polysaccharide CSPA (molecular weight, 1.394 × 10^5^ Da) by super hydrogel 500 gel filtration chromatography. The drug experiment showed that CSPA significantly reduced the blood sugar levels in streptozotocin-induced diabetic rats. In addition, Suvdmaa TuvaanJava et al. [22] purified two types of *Cynomorium songaricum* polysaccharides using Sephadex G-75 and G-50 continuous column chromatography, and their molecular weights were determined to be 3.7 × 10^4^ and 1.0 × 10^4^ Da, respectively. Both were found to exert a strong inhibitory effect on HIV infection in MT4 cells. The aim of this study was to obtain CSP and to determine its immunoregulatory activities.

Thus, to examine the immunological activity of *Cynomorium songaricum* polysaccharides, T and B lymphocytes and RAW264.7 macrophages were employed as the cell models in this study. The RAW264.7 cell line is a frequently used cellular model in the research of phytopolysaccharide activity, because it has been well described in terms of metabolism, phagocytosis, and macrophage-mediated immunity [23]. It was discovered that the primary way in which polysaccharides contribute to immunity is by stimulating complement receptors, T and B lymphocytes, macrophages, and the generation of cytokines, including IL-6 and tumor necrosis factor (TNF)-α. Key lymphocytes such as B and T cells are the primary carriers of the immune response in the body, and also serve as markers of the capacity and state of the immune system [24,25]. Meanwhile, T cells are crucial for the generation of a wide variety of antibodies and for the immune response, and B cells mainly perform humoral immunity and are capable of producing a wide range of antibodies. T cells can control the immune response brought on by antigens, whereas B cells are primarily in charge of humoral immunity and are capable of producing various antibodies. In this study, *Cynomorium songaricum* polysaccharides exerted immune effects by promoting the proliferation of T and B lymphocytes and macrophages.

In the meantime, research was conducted to explore the CSP-III signaling pathway in cells, and the effect of CSP-III on the expression of MAPK signaling pathway-related proteins was investigated. Notably, the MAPK signaling pathway is an important signaling system that helps convey extracellular signals to intra-cellular proteins. It controls a variety of cellular functions, such as differentiation, apoptosis, proliferation, and stress responses [26]. Additionally, the ERK-MAPK pathway is one of the most significant mechanisms for cellular proliferation, and the expression of this system determines development [27]. Numerous pro-inflammatory and stress-related stimuli, like interleukins, TNF-α, activating cytokine receptors, and Toll-like receptors, activate the P38-MAPK pathway. Consequently, this pathway is a possible target in inflammatory diseases since it is involved in distinct biological reactions other than inflammation [28]. Abnormalities in the JNK-MAPK signaling pathway have been suggested to be linked to many immunological illnesses and malignancies. This pathway governs a wide range of biological activities, including cell proliferation, differentiation, survival, apoptosis, and inflammation [29].

On the basis of in vitro experiments, a CTX-induced immunosuppressive mouse model was adopted. CTX is a medication frequently used to create the immunosuppressive animal model, since it can decrease both humoral and cellular immunity. Research has indicated that CTX modifies the intestinal flora, harms the gastrointestinal tract mucosa and lowers immunity. Furthermore, *Cynomorium songaricum* polysaccharides are discovered to improve immune organ atrophy in CTX-exposed mice, increase the IgG and IgM levels in the body, and activate the MAPK signaling pathway, thereby enhancing the body immune function by regulating both humoral and cellular immunity. In the spleen lymphocyte proliferation experiment, compared with the blank group, the low-dose group of Cynomorium songaricum polysaccharides inhibited T lymphocyte proliferation in vitro. However, subsequent experiments found that CSP—III had a significant promoting effect on the lymphocyte transformation rate in mice, suggesting that the low-dose group of *Cynomorium songaricum* polysaccharides may need to go through the in vivo and in vitro metabolism to participate in lymphocyte differentiation and maturation [30].

The intestine is a major organ for digestion and absorption in the body, which also serves as a vital line of defense against harmful substances and microbes that attempt to enter the body. An increasing number of studies have demonstrated a similar regulatory mechanism between intestinal flora and immune function and polysaccharides. By conducting the 16S rDNA high-variable region assay, alterations in the intestinal flora of immunosuppressive mice treated with *Cynomorium songaricum* polysaccharides were identified. The disruption of intestinal flora brought on by CTX was reversed in the high-dosage group, and the OTU diversity and flora structure were returned to normal levels in mice. Collectively, *Cynomorium songaricum* polysaccharides can either directly activate the appropriate immune pathway, maintain bacterial homeostasis to regulate intestinal homeostasis, and enhance the organism’s immune function, or increase the relative abundances of certain bacteria, such as Bacteroides immitis, Desmodium, and Actinobacteria.

## 4. Materials and Methods

### 4.1. Materials

Our samples were wild products collected from Hotan (37°12′ N/79°92′ E), Xin Jiang, China, which were identified by Professor Zhang Hui from the University of Traditional Chinese Medicine as the dry and fleshy stems of *Cynomorium songaricum* (*Cynomorium songaricum* Rupr.). The dry and fleshy stems were flat cylindrical, slightly curved, 5–15 cm in length and 1.5–5 cm in diameter. The surface was brown and rough, with obvious longitudinal grooves and irregularity. The samples complied with the specifications of the Pharmacopoeia of the People’s Republic of China 2020.

RAW264.7 macrophages were obtained from our laboratory, meanwhile, 60 5-week-old SPF-grade BALB/c mice weighing 18 ± 2 g (male to female ratio, 1:1) were also obtained for the experiments.

### 4.2. Reagents

Glucose (Glc), glucuronic acid (GlcA), fucose (Fuc), galactose (Gal), galacturonic acid (GalA), arabinose (Ara), xylose (Xyl), rhamnose (Rha), mannose (Man) control, sodium tetraborate, carbazole, cyclophosphamide (CTX), and 1-phenyl-3-methyl-5-pyrazolone (PMP) were purchased from McLean Biochemistry & Technology Co., Ltd. (Shanghai, China). Analytically pure reagents including trichloromethane, n-butanol, and concentrated sulphuric acid were bought from Sinopharm (Beijing, China). Levamisole was provided by Huazu. Sephadex G-100 and DEAE-52 cellulose were acquired from Shanghai Yuan Ye Bio-Technology Co., Ltd. (Shanghai, China). The Caulmers Brilliant Blue G-250 was provided by Biosahrp. Lipopolysaccharide, Concanavalin A, Dialysis Bag, and Macroporous Adsorbent Resin D101 were acquired from Beijing Solarbio Science & Technology Co., Ltd. (Beijing, China). The Mouse Splenic Lymph Isolate Kit was purchased from TBD. Rabbit Anti-P-p38 Monoclonal Antibody Rabbit, Mouse Anti p38, JNK Monoclonal Antibody, Anti-P-JNK Polyclonal Antibody, Goat Anti-Rabbit IgG, and Goat Anti-Mouse IgG were purchased from Proteintech Group, Inc. (Wuhan, China). Mouse anti-α-Tubulin monoclonal antibody, Rabbit anti-ERK, and P-ERK monoclonal antibody were obtained from abclonal. The Mouse Immunoglobulin G Antibody ELISA Kit and Mouse Immunoglobulin M Antibody ELISA Kit were purchased from Jiangsu Enzyme Immune Industry Co., Ltd. (Yancheng, China).

### 4.3. Instruments

A cryogenic freezing high-speed centrifuge (Thermo Fisher Scientific Co., Ltd. Waltham, MA, USA), an ultraviolet-visible spectrophotometer (Beijing Pudian General Limited Liability Company, Beijing, China), an Agilent 1200 High-Performance Liquid Chromatograph (Agilent, Santa Clara, CA, USA), and an Alpha1-2LDplus model vacuum freeze dryer (Christ, Germany, Osterode, Germany) were used in this study.

### 4.4. Isolation and Purification of Polysaccharides

First of all, the *Cynomorium songaricum* stems were dried at 40 °C, later crushed and passed through a 60-mesh sieve. Thereafter, the materials were defatted for 2 h using a 95% ethanol reflux according to the material–liquid ratio and then dried at room temperature for later use.

The extraction process of CSP was as follows, degreasing powder → water bath heating → centrifugation, supernatant combination → alcohol precipitation → washing precipitation → protein removal → dialysis → freeze-drying → CSP.

To create the purified *Cynomorium songaricum* polysaccharide, also known as CSP-I, a suitable amount of crude polysaccharide extracted from *Cynomorium songaricum* was collected, diluted in 10 mL distilled water, and enriched with the macroporous adsorbent resin D101.

A DEAE-52 cellulose column was used for the consecutive elution of 10 mL aqueous solution of CSP-I (20 mg/mL) with distilled water and NaCl solutions at varying concentrations [31]. Thereafter, 80 tubes were collected over the course of 10 min at a flow rate of 0.5 mL/min. The elution curve was then plotted with the number of tubes as the horizontal coordinate, whereas the measured A-value was the vertical coordinate, so as to determine the total sugar content in each separated tube at a wavelength of 490 nm. To obtain the purified *Cynomorium songaricum* polysaccharide CSP-II, the eluate was collected, dialyzed under running water for 48 h, and finally freeze-dried under vacuum.

Afterwards, the samples were carefully put into the Sephadex G-100 gel column, followed by 2 h of equilibration prior to elution with ultrapure water. The flow rate was controlled, and 4 mL eluent was collected in each tube after the CSP-II was precisely weighed and dissolved in 4 mL of distilled water [32]. To acquire the purified *Cynomorium songaricum* polysaccharide CSP-III, the eluate was collected, concentrated, and lyophilized after the elution curve was constructed. Eventually, the phenol sulphate technique was used to determine the polysaccharide content.

### 4.5. Analysis of Monosaccharide Composition 

After carefully weighing the appropriate amounts of Man, Fuc, GlcA, GalA, Glc, Gal, Xyl, Rha, and Ara control, water was added to prepare a single standard solution with a concentration of 1.0 mg/mL. To create a mixed standard solution with nine sugars at a concentration of roughly 0.1 mg/mL, 1.0 mL of each single standard solution was pipetted into a 10 mL measuring flask. Later, 500 μL of the blended standard solution was accurately extracted, then 0.25 mol/l NaOH solution (75 μL) and 0.5 mol/l PMP methanol solution (150 μL) were added in succession and blended thoroughly, followed by 90 min reaction under 70 °C. After cooling to room temperature, 0.25 mol/l of HCL solution (75 μL) was added. Later, 1 mL of trichloromethane was added into the extract, the bottom layer of the organic layer was scraped out, and the extraction was repeated thrice. After removing the top layer of the solution, the solution was tested. The chromatographic conditions are shown in Table 2.

#### Preparation of Test Solutions

To be specific, 5 mg of CSP-III samples was precisely weighed and added into the trifluoroacetic acid solution (4 mol/L), followed by sealing, 4 h of hydrolysis at 100 °C, withdrawing, and blowing dry under nitrogen. Later, the methanol solution was added to remove excess trifluoroacetic acid, and the sample was then dissolved with water, followed by derivatization [33]. 

### 4.6. Molecular Weight Determination via High-Performance Gel Molecular Exclusion Chromatography 

#### 4.6.1. Standard Curve Preparation and Sample Determination

The dextran control was precisely weighed to obtain its molecular weight, then a mobile phase was added to obtain a solution with a concentration of 5 mg/mL. After sample injection, the standard curve was drawn, where the horizontal coordinate represents the retention time of the chromatographic peak, while the vertical coordinate stands for the logarithmic value of the standard molecular weight.

#### 4.6.2. Preparation of Test Solutions

In brief, 5 mg CSP-III was weighed precisely, added into the 1 mL mobile phase, mixed sufficiently, and placed aside after being passed through a 0.45-μm filter membrane.

#### 4.6.3. Chromatographic Conditions

The sample was injected in line with the chromatographic conditions displayed in Table 3 and the molecular weight was calculated from the retention time of the peaks [34].

### 4.7. In Vitro Experiments

#### 4.7.1. Splenocyte Proliferation Assay

##### Culture of Mouse Splenic Lymphocytes

After the spleen was dissected, splenocytes were separated using a mouse splenic lymphatic isolation kit on a highly clean bench and deposited in a Petri plate under aseptic circumstances [35]. Thereafter, the Petri dish was filled with 5 mL homogenizing washing solution before being ground, and the grinding solution was collected for 10 min of centrifugation at 450 g to collect the supernatants. Following cell resuspension in the sample diluent, an equivalent volume of isolation solution was added and later centrifuged for 25 min. Afterwards, a ringed layer of milky-white lymphocytes was extracted from the stratified liquid after stratification. The washing solution was then added and, after 10 min of centrifugation at 400 g, the supernatants were collected. Later, the washing solution was added again, cells were mixed and centrifuged at 400× *g* for 10 min twice. The supernatants were discarded, and then cells were resuspended with RPMI-1640 cell culture medium. After resuspension, the cell density was titrated to 2 × 10^6^ cells/mL and the cells were subsequently seeded onto the 96-well plates.

##### T-Lymphocyte Proliferation Assays

In these assays, the blank group, ConA control group, cell control group, and drug administration group (final concentrations of 25, 50, 100, 200, and 400 μg/mL) were set up. Subsequently, an equal volume of ConA solution (10 μg/L) was added into each well, and the cells were incubated at a constant temperature with 5% CO_2_ at 37 °C for 44 h. At 4 h after the addition of the CCK-8 reagent, the optical density (OD) value in each group was calculated [36].

##### B-Lymphocyte Proliferation Assays

The blank group, LPS control group, cell control group, and drug administration group were set up. Afterwards, an equal volume of LPS solution (5 μg/mL) was added into each well to incubate for 44 h at 37 °C with 5% CO2. At 4 h after CCK-8 addition, the OD value of each group was determined [36].

#### 4.7.2. RAW264.7 Macrophage Proliferation

RAW264.7 macrophages were concentrated to 2 × 10^5^ cells/mL, seeded onto the 96-well plates, and incubated for 24 h at 37 °C with 5% CO_2_. The final concentrations of 25, 50, 100, 200, and 400 μg/mL were used, meanwhile, the blank, control, positive (LPS) and drug-dosing groups were established. After incubation for 24 h, CCK-8 was added, then the OD value of each group was recorded after 40 min, and the cell proliferation rate was computed [37].

#### 4.7.3. Impact of CSP-III on the Expression of MAPK Signaling Pathway-Related Proteins

After adjusting the concentration of RAW264.7 macrophages to 2 × 10^5^ cells/mL, the cells were inoculated onto the 96-well plates and incubated in an incubator at 37 °C with 5% CO_2_ for 24 h. The liquid was later discarded, and three groups, including the blank, positive (LPS), and drug delivery group (with final concentrations of 100, 200, and 400 μg/mL) were set up. At 12 h after drug administration, the cellular proteins were extracted. Finally, a Western blotting assay was conducted to ascertain the protein expression levels of p38, p-p38, JNK, p-JNK, ERK, and p-ERK (Table 4).

### 4.8. In Vivo Experiments

#### 4.8.1. Animals

Sixty 5-week-old SPF-grade BALB/c mice (male to female ratio, 1:1; body weight, 18 ± 2 g) were randomly assigned into 6 groups, including a blank group (BG), a cyclophosphamide model group (MG), a levamisole-positive drug group (PG), a CSP-III low-dosage group (SL), a CSP-III medium-dosage group (SM), and a CSP-III high-dosage group (SH). At 7 days after successful modeling, the corresponding drugs were given to the corresponding drug groups through gavage administration for 21 days, and a normal dosage was subcutaneously injected into the positive drug group. Meanwhile, an equivalent volume of physiological saline was administered in both the model group and the normal control group through gavage.

#### 4.8.2. Splenic Lymphocyte Proliferation Assay

As described in 4.7.1.1, splenic lymphocytes were prepared. Cells were grown in an incubator with 5% CO_2_ at 37 °C for 4 h, after which, 100 μL of either LPS or ConA solution was added to the wells. The total volume of each well was 200 μL, and the final concentrations of LPS and ConA were 10 μg/mL and 5 μg/mL, respectively. Eventually, the OD value was observed at 570 nm.

#### 4.8.3. Identification of the T-Lymphocyte Subset by Flow Cytometry

The lymphocyte preparation process is detailed in 4.7.2. To prepare a 1 × 10^5^~1 × 10^7^ cell suspension, 500 μL of ice-cold PBS containing 10% FBS was added to resuspend the cells. Later, Fc Block was added for Fc receptor closure, and the cells were then kept closed for 10 min at 4 °C. Following cell dispension into the flow tubes, the cells were centrifuged for 3 min at 200 r/min, the supernatants were thrown away and the monoclonal antibodies against CD8-PE, CD4-FITC, and CD3-APC were added. Following the addition of CD8-PE, CD4-FITC, and CD3-APC, the cells were thoroughly mixed and incubated at 4 °C for 30 min under light protection. To eliminate any remaining free fluorescent antibodies, the cells were washed with ice-cold PBS buffer (200 r/min for 3 min) thrice. Finally, the cells were resuspended in ice-cold PBS buffer, filtered with a 200-mesh membrane and placed aside [35].

#### 4.8.4. ELISA Technique for Detecting Serum IgG and IgM Levels in Mice

Blood was drawn from mouse eyes under sterile conditions into the sterile centrifuge tubes with clear labels, spun at 4 °C for 20 min, then the supernatants were carefully removed and kept at −80 °C. The serum IgG and IgM levels were determined in line with the kit’s instructions.

#### 4.8.5. HE Staining

In brief, 4 spleen and mesenteric lymph nodes randomly chosen from each group were fixed with 4% paraformaldehyde, and the trimmed tissues were later dehydrated with gradient ethanol, encased in paraffin wax and divided into parts (3 m in thickness). The sections were then stained, placed under a microscope, and magnified to the appropriate level for microscopic examination. Finally, images were captured and analyzed [38].

#### 4.8.6. Western Blotting Assays

Total proteins were extracted from mouse spleen tissues, and the protein content was quantified after determining the concentration of each group. Then, the protein expression levels of P38, p-p38, JNK, p-JNK, ERK, and p-ERK were determined by Western blotting assay.

#### 4.8.7. Intestinal Flora

Mouse fecal samples were collected, from which genomic DNA was extracted using 16s rRNA sequencing technology, and the intestinal microorganisms were detected in the fecal samples of each group of mice.

### 4.9. Statistical Analysis

Data were expressed as mean ± standard deviation (S.D.). The one-way ANOVA and Dunn’s multiple comparison post hoc tests were performed using GraphPad Prism 9.0.0 (GraphPad-Software Inc., San Diego, CA, USA) to calculate and compare biochemical indices among different groups. The *p*-value < 0.05 stood for statistical significance.

## 5. Conclusions

In this study, CSP-III was isolated from *Cynomorium songaricum*, and the monosaccharide composition included Man, GlcA, GalA, Rha, Glc, Gal, and Ara, with a molecular weight of 4,018,234 Da. According to our in vitro experimental results, within a certain concentration range, CSP-III promoted the proliferation of mouse macrophages and T and B lymphocytes, while activating the JNK MAPK signaling pathway. Through the study of CTX-induced immunosuppressive mouse models, it was found that CSP-III improved CTX-induced atrophy of immune organs in mice, increased the levels of IgG and IgM in the body, and activated the MAPK signaling pathway, thereby enhancing the body’s immune function by regulating both humoral and cellular immunity. In the analysis of intestinal flora. CSP-III was able to maintain intestinal homeostasis, improve intestinal flora, and enhance immune function by regulating changes in intestinal flora. In summary, we have demonstrated that the polysaccharide CSP-III isolated from *Cynomorium songaricum* has strong immune regulatory activity, and the mechanism of its immune enhancement may involve the regulation of MAPK signaling pathway and intestinal flora. Findings in the present study provide a theoretical basis for further research on the development of functional foods from *Cynomorium songaricum* and for the scientific interpretation of the traditional efficacy of Cynomorii Herba.

However, the structure of *Cynomorium songaricum* polysaccharides and their other functions should be further investigated.

## Figures and Tables

**Table 1:** Polysaccharide Content.

Sample	Total Sugar Content (%)
CSP-II-1	7.28
CSP-II-2	20.07
CSP-II-3	79.50
CSP-II-4	36.22
CSP-II-5	5.87

**Table 2:** Chromatographic conditions.

	Condition
Column temperature	40 °C
Detection wavelength	250 nm
Velocity of flow	0.8 mL/min
Sample injection volume	20 μL
Mobile phase A	Phosphate buffer solution (pH 6.8)—acetonitrile (85:15)
Mobile phase B	Phosphate buffer solution (pH 6.8)—acetonitrile (60:40)

**Table 3:** Chromatographic conditions.

	Condition
Chromatographic column	SRT SEC-100 (7.8 mm × 300 mm) gel column
Detector	Oscillometric refractive detector
Column temperature	35 °C
Velocity of flow	0.5 mL/min
Sample injection volume	20 μL
Mobile phase	Ultrapure water

**Table 4:** Antibodies used in the experiments.

Antibody Name	Dilution Ratio
Mouse Anti-p38 monoclonal antibody	1:1000
Rabbit Anti-P-p38 monoclonal antibody	1:1000
Mouse Anti-JNK monoclonal antibody	1:1000
Rabbit Anti-P-JNK polyclonal antibody	1:1000
Rabbit Anti-ERK monoclonal antibody	1:1000
Rabbit Anti-P-ERK monoclonal antibody	1:1000
Mouse Anti-α-Tubulin monoclonal antibody	1:5000
Goat Anti-Rabbit IgG	1:6000
Goat Anti-Mouse IgG	1:6000

## Data Availability

Data are contained in the article.

## Abbreviations

CTX	Cyclophosphamide
CSP-I	Purification of polysaccharides using macroporous adsorption resin D101
CSP-II	Purification of polysaccharides using DEAE-52 cellulose column
CSP-III	Purification of polysaccharide by Sephadex G-100 gel column chromatography
PMP	1-phenyl-3-methyl-5-pyrazolone
Mw	Mass average molar mass
PDI	Polymer dispersity index
HPLC	High Performance Liquid Chromatography
MAPK	Mitogen-activated protein kinase
P38	p38 MAPK
ERK	Extracellular regulated protein kinases
JNK	c-Jun N-terminal kinase
CCK-8	Cell Counting Kit-8
LPS	Lipopolysaccharide
Ig	Immunoglobulin
IL	Interleukin
ConA	Concanavalin A
PBS	Phosphate Buffer
TNF	Tumor necrosis factor
SCFAs	Short chain fatty acid
